# Dual-Color Expansion Microscopy of Membrane Proteins Using Bioorthogonal Labeling

**DOI:** 10.1021/acs.nanolett.5c05301

**Published:** 2026-01-22

**Authors:** Steven Edwards, Birthe Meineke, Sebastian Bauer, Hans Blom, Simon Elsässer, Hjalmar Brismar

**Affiliations:** † Science for Life Laboratory, 7655 KTH Royal Institute of Technology, 171 21 Solna, Sweden; ‡ Science for Life Laboratory, Karolinska Institute, 171 21 Solna, Sweden

**Keywords:** Expansion microscopy, Noncanonical amino acids, Super-resolution microscopy, Bioorthogonal chemistry, Linkage error, Site-specific labeling

## Abstract

With recent advances in fluorescence microscopy, resolution is often limited by the size of the label and the resulting linkage error, rather than the microscope itself. Site-specific incorporation of noncanonical amino acids (ncAAs) combined with bioorthogonal click chemistry provides a powerful tool for fluorescent protein labeling, overcoming the spatial uncertainty inherent to antibody-based probes. Here, we present a method to further improve labeling precision by combining ncAA labeling with expansion microscopy (ExM) for dual-color super-resolution imaging. After optimizing labeling procedures and fluorophore selection, we visualize and resolve the nanoscale distribution of Na,K-ATPase α_1_ and β_1_ subunits in expanded HEK 293T cells. We validate our approach by super-resolution STED imaging of the ncAA labeled β_1_ subunit in unexpanded cells. This work presents a strong framework for multiplexed, high-resolution imaging, suggesting that ncAA labeling combined with ExM enables biological imaging at the nanometer scale.

## Graphical abstract

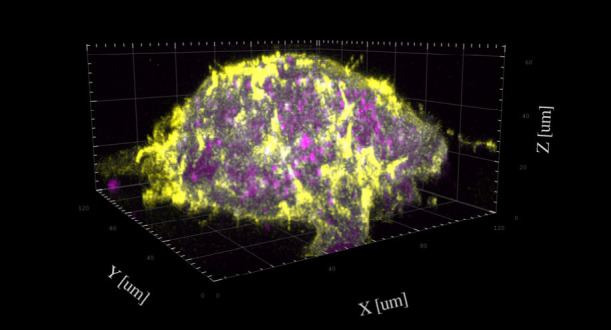

Deciphering the nanoscale organization of membrane proteins is fundamental to understanding cell biology, yet this remains a significant technical challenge due to the inherent difficulty in achieving labeling without spatial distortions and access to super-resolution microscopy methods. A key protein of interest is the Na,K-ATPase (NKA), a ubiquitous integral membrane protein essential for maintaining ion gradients in virtually all eukaryotic cells.^1^ While NKA is known to be a heterodimer composed of α and β subunits, its higher-order spatial arrangement within the plasma membrane is not fully understood.^2–6^ Determining whether NKA functions as individual heterodimers or as part of larger oligomeric complexes requires imaging technologies that can resolve molecules at a scale well below the diffraction limit of light.^7^

To address this challenge, super-resolution microscopy techniques, such as STED and SMLM, have become valuable tools. However, the ultimate resolution of these methods is dependent on the labeling strategy.^8^ Traditional antibody based immunolabeling can introduce a “linkage error” of tens of nanometers due to the physical size of the antibodies, obscuring the true location of the target protein.^9^ Self-labeling tags like SNAP and Halo have been introduced as attractive alternatives, but there are reports of inconsistent labeling efficiency using those approaches due to varying cellular environments.^10^

A more precise alternative is the site-specific incorporation of noncanonical amino acids (ncAAs) via genetic code expansion (GCE).^11^ This technique allows for the placement of a small chemical handle at a specific site within a protein. A subsequent bioorthogonal “click chemistry” reaction can then attach an organic fluorophore with minimal linkage error, enabling a more precise representation of the protein’s location.^12^

In parallel, expansion microscopy (ExM) has emerged as an attractive and accessible super-resolution technique.^13^ By physically enlarging the biological specimen within a swellable hydrogel, ExM makes it possible to achieve nanoscale resolution using conventional, diffraction-limited microscopes.^14,15^

We have combined these technologies and present here a workflow that integrates GCE-based bioorthogonal labeling with ExM for dual-color super-resolution imaging. By targeting the α_1_ and β_1_ subunits of NKA, we visualize the enzyme in expanded HEK 293T cells, providing new insights into its nanoscale distribution.^7,16,17^ We further validate our approach through a qualitative comparison with STED microscopy. This work establishes a robust framework for multiplexed, high-resolution imaging and demonstrates how the synergy between ncAA labeling and ExM can advance biological nanoscale imaging.

Genetic code expansion via stop codon suppression allows the expression of ncAA containing proteins. Introduction of an orthogonal tRNA/aminoacyl-tRNA synthetase pair and its cognate ncAA recodes the stop codon and can lead to incorporation of the ncAA in that position, instead of translation termination (Figure 1A). We used genetic code expansion with amber (TAG), or ochre (TAA) stop codon suppression to introduce ncAAs with clickable functional groups into the extracellular region of the NKA β_1_ and α_1_ subunits. For ochre codon suppression we used *M. mazei* (*Mma*) pyrrolysyl-tRNA (tRNA^Pyl^) variant M15_UUA_/pyrrolysyl-tRNA synthetase (PylRS) pair which efficiently incorporates N-propargyl-l-lysine (ProK). ProK can be specifically labeled with a picolyl azide modified dye using Cu­(I)-catalyzed azide–alkyne cycloaddition (CuAAC).^18^

**Figure 1:**
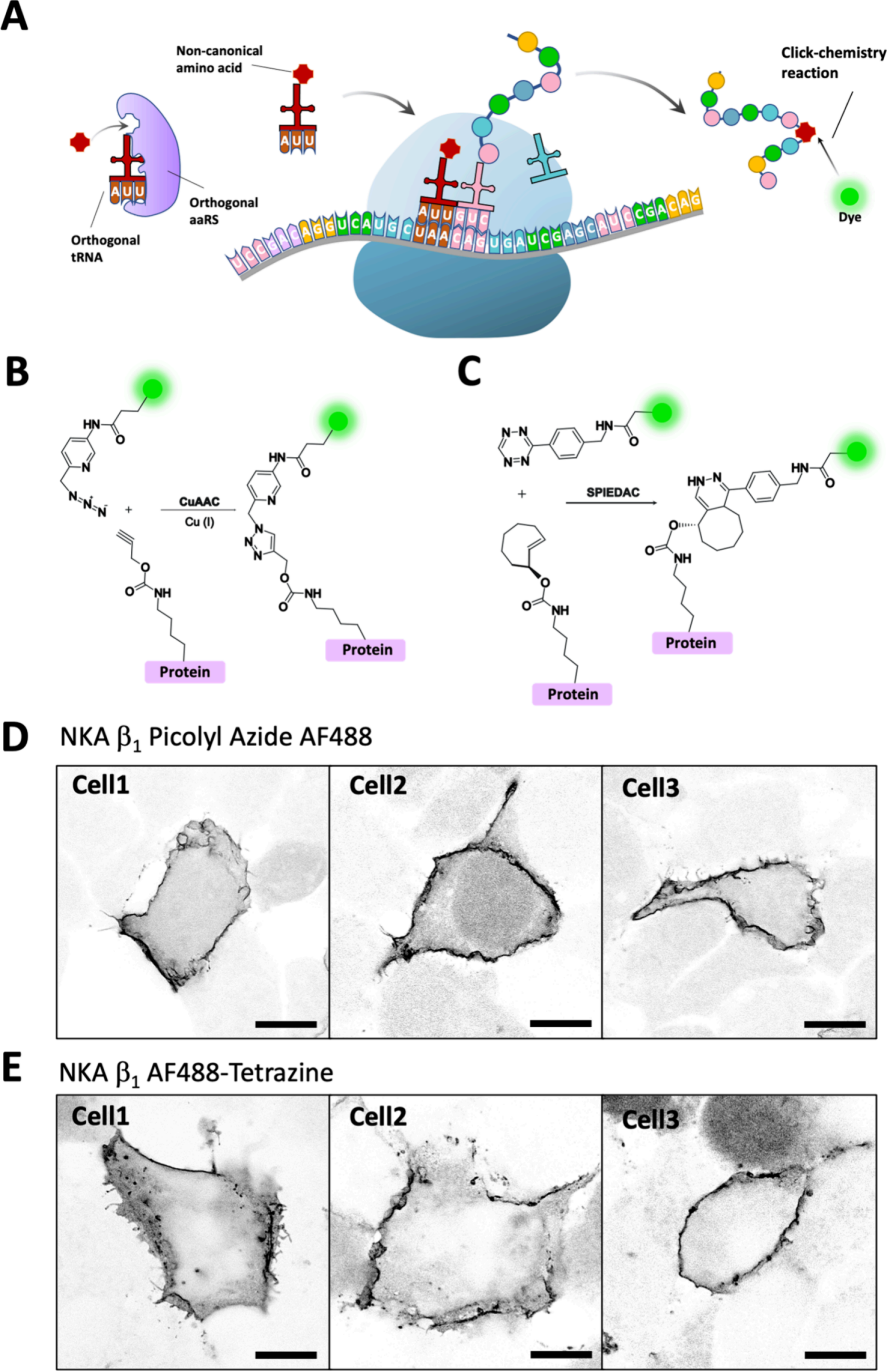
Genetic code expansion uses an orthogonal tRNA/synthetase pair to incorporate a noncanonical amino acid into the target protein (A), allowing selective fluorescent labeling via click chemistry. The figure illustrates the chemical schemes for CuAAC (B) and SPIEDAC (C). Confocal microscopy confirms successful labeling of NKA β1 L64ProK via CuAAC with AF488 picolyl azide (D) and NKA β1 L64TCO*K via SPIEDAC with AF488-tetrazine (E), in fixed HEK293T cells. Scale bars: 10 μm.

To demonstrate successful incorporation of the ncAA and transport of NKA to the plasma membrane, HEK293T cells were cotransfected with NKA β_1_ L64TAA mutant, the M15_UUA_/*Mma*PylRS pair, and an NKA α_1_ WT to improve membrane insertion of the protein. The cell culture media was supplemented with ProK and cells were allowed to grow for 48 h before click-labeling with AF488-picolyl azide, fixation and imaging using confocal microscopy. Fluorescent signal was detected in the plasma membrane of the cells (Figure 1B), indicating expression, membrane insertion and click labeling of NKA β_1_ L64ProK.

We designed and produced a NKA β_1_ L64TAG mutant which was transfected with its amber suppressor hyb*/*G1*PylRS*YA* pair for TCO*K incorporation. TCO*K can be labeled by SPIEDAC click-chemistry with tetrazine modified dyes. The cell culture media was supplemented with TCO*K and cells were allowed to grow 48 h before labeling with AF488-tetrazine, fixation and imaging using confocal microscopy. Once again, fluorescent signal was detected in the plasma membrane of the cells (Figure 1C). The AF488 dye used for click-labeling is membrane impermeable and should therefore only label the ncAA that are located extracellularly.

To validate the specificity of ncAA incorporation and ensure the expression of full-length proteins, we performed Western blot analysis of cell lysates. We observed a distinct band corresponding to the full-length NKA α_1_ and β_1_ subunits only when cells were supplemented with the ncAA. In control samples lacking the ncAA, the full-length protein was not detected, confirming the robustness of the suppression system (Figure S1 in the Supporting Information).

To investigate the nanoscale distribution of the NKA β_1_ subunit, we first used STED super-resolution microscopy. Given that STED imaging requires bright and highly photostable fluorophores, we labeled the β_1_ subunit with either Abberior STAR 635-tetrazine or Abberior STAR RED-tetrazine dyes. As both dyes are membrane-impermeable, labeling was successfully restricted to β_1_ subunits located in the plasma membrane of the cells (Figure 2A,B). This is the first demonstration of successful use of STED optimized dyes for click-labeling. The resulting STED images showed a nonhomogenous distribution of NKA β_1_ at the cell membrane, which is consistent with previous reports.^7^

**Figure 2:**
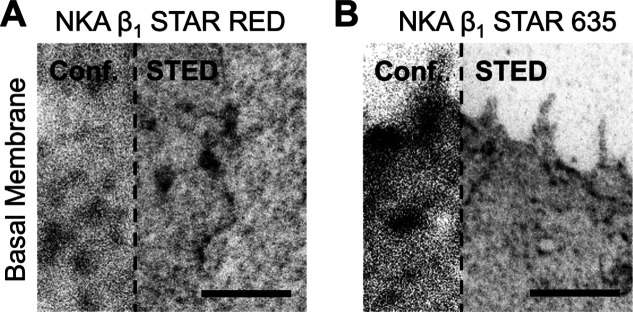
Super resolution STED microscopy of NKA β_1_ in the plasma membrane. SPIEDAC labeling of the NKA β1 L64TCO*K on the surface of HEK239T cells, fixed and imaged using confocal and STED microscopy. Abberior STAR RED tetrazine (A) and Abberior STAR 635 tetrazine (B) both showed labeling in the basal membrane. Scale bars: 2 μm.

As an alternative approach to achieve super-resolution imaging, we utilized expansion microscopy (ExM), a technique that allows for nanoscale imaging on diffraction-limited microscopes. For this method, HEK293T cells were cotransfected to express NKA β_1_ L64ProK, which was subsequently click-labeled with AF488-picolyl azide. Proteins in the sample were then cross-linked into a swellable hydrogel. Following denaturation in an SDS containing buffer, the hydrogel was expanded in deionized water, resulting in an isotropic physical expansion of approximately 4.3-fold (Figure 3A).

**Figure 3:**
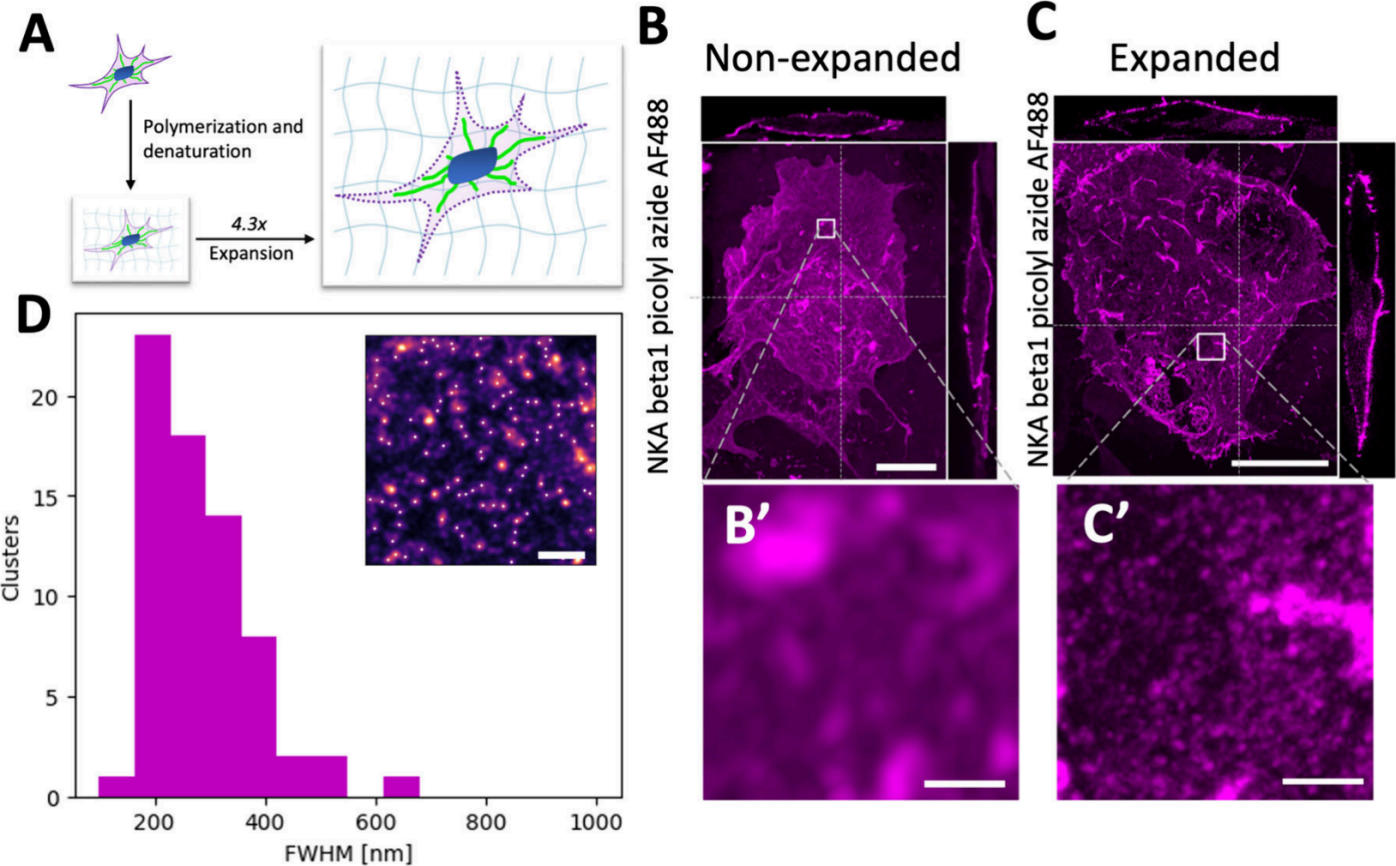
Expansion microscopy enables super resolution imaging of NKA β_1_ in the plasma membrane on diffraction limited microscopes. Schematic representation of the expansion microscopy process, a fixed cell is embedded into a swellable polyelectrolyte gel and denatured at 95 °C in an SDS containing buffer. The gel is immersed in deionized water to fully expand approximately 4.3-fold (A). Confocal z-stack (MIP) of nonexpanded HEK293T cells expressing NKA β1 L64ProK labeled with AF488-picolyl azide by CuAAC. Orthogonal views are shown for the positions marked with a dashed line (B). Confocal z-stack (MIP) of expanded HEK293T cells expressing NKA β1 L64ProK labeled AF488-picolyl azide by CuAAC (C). Distribution of cluster sizes in the expanded cells. Clusters are identified by a spot analysis finding local intensity maxima and fitting to 2D Gaussian. Inset is a magnified view of a cell with identified local intensity maxima marked in white (D). Scale bars: (B) and (C) 10 μm; (B′) and (C′) 1 μm (C and C′ are adjusted for expansion); (D) 2 μm.

We imaged both fixed, unexpanded cells (Figure 3B) and expanded cells (Figure 3C) using an Airyscan microscope using a 40 × 1.2 NA water immersion objective. In the expanded samples, the nonhomogenous distribution of NKA in the apical membrane was more clearly resolved than in the unexpanded samples.

To quantitatively describe the NKA organization, we analyzed the labeled protein distribution in our ExM images (Figure 3D). This analysis identified distinct aggregates in the expanded gel with sizes ranging from 150 to 600 nm, with a mean diameter of 286 nm. The lower bound of this range (150 nm) is constrained by the diffraction limit of our imaging setup. After correcting for the measured 4.3-fold expansion factor, these values correspond to a physical size range of ∼35–140 nm. Taken into consideration that this number is still diffraction limited, it is in good agreement with previous super-resolution studies using SMLM and STED, which reported NKA cluster sizes in the 20–50 nm range.^7,16,17^

We next tested the possibility of performing two-color labeling by combining a pre-expansion labeling step with a postexpansion one. It is possible to perform a first CuAAC labeling reaction with AF488-picolyl azide before fixation and a second reaction with AF647-picolyl azide after the sample has been denatured in the gel. Because the expansion protocol permeabilizes the cell, the pre-expansion AF488 labeling is limited to the plasma membrane, whereas the postexpansion AF647 labeling targets both extracellular and intracellular ncAAs (Figure S2).

To validate the specificity of this intracellular, postexpansion labeling, we performed a control experiment in which NKA β_1_ WT or NKA β_1_ L64TAA was transfected together with the tRNA/aaRS pair for ochre suppression. Gels were labeled after denaturation with AF488-picolyl azide. Both cell types exhibited similar levels of intracellular fluorescence after postexpansion labeling, indicating that this signal was nonspecific (Figure S3). Furthermore, we tested if the SPIEDAC labeling chemistry could be performed after denaturation but found that its reactive handle (TCO*K) was no longer reactive after the gelation and denaturation process (not shown).

SPIEDAC and CuAAC click-chemistries can be combined for distinct two-color fluorescent labeling of ProK and TCO*K site-specifically incorporated into cell membrane proteins.^19^ For this we combined ochre suppression by M15_UUA_/*Mma*PylRS for ProK incorporation with amber suppression by hyb*_CUA_/*G1*RS YA for axial *trans*-cyclooct-2-ene-l-lysine (TCO*K) incorporation. We have previously used this approach to label NKA subunits α_1_ and β_1_ with two fluorophores for quantification of NKA by FRET/FCS.^3^ We now used this approach to do two color expansion microscopy by cotransfection of HEK293T cells with NKA α_1_ T121TAG and NKA β_1_ L64TAA together with both tRNA/aaRS pairs. AF488-picolyl azide was combined with Abberior STAR 635-tetrazine to spectrally separate the fluorescence emission. Both dyes were preserved throughout the expansion process and membrane expression of the two fluorescently labeled subunits could be detected (Figure 4A and A′). Unlike Abberior STAR 635-tetrazine, AF647-tetrazine was quenched during the expansion process (data not shown). Confocal Airyscan images of the lateral membrane revealed the distribution of α_1_ and β_1_ subunits with high contrast. We also measured the intensity of labeled α_1_ and β_1_ subunits along the highlighted lateral membrane region (Figure 4C), evaluated as normalized intensity ratios (*I* – *I*_min_)/(*I*_max_ – *I*_min_) and plotted a line profile (Figure 4D). This visualization of the heterodimeric NKA is, to our knowledge, the first example of two-color super resolution expansion microscopy with GCE.

**Figure 4:**
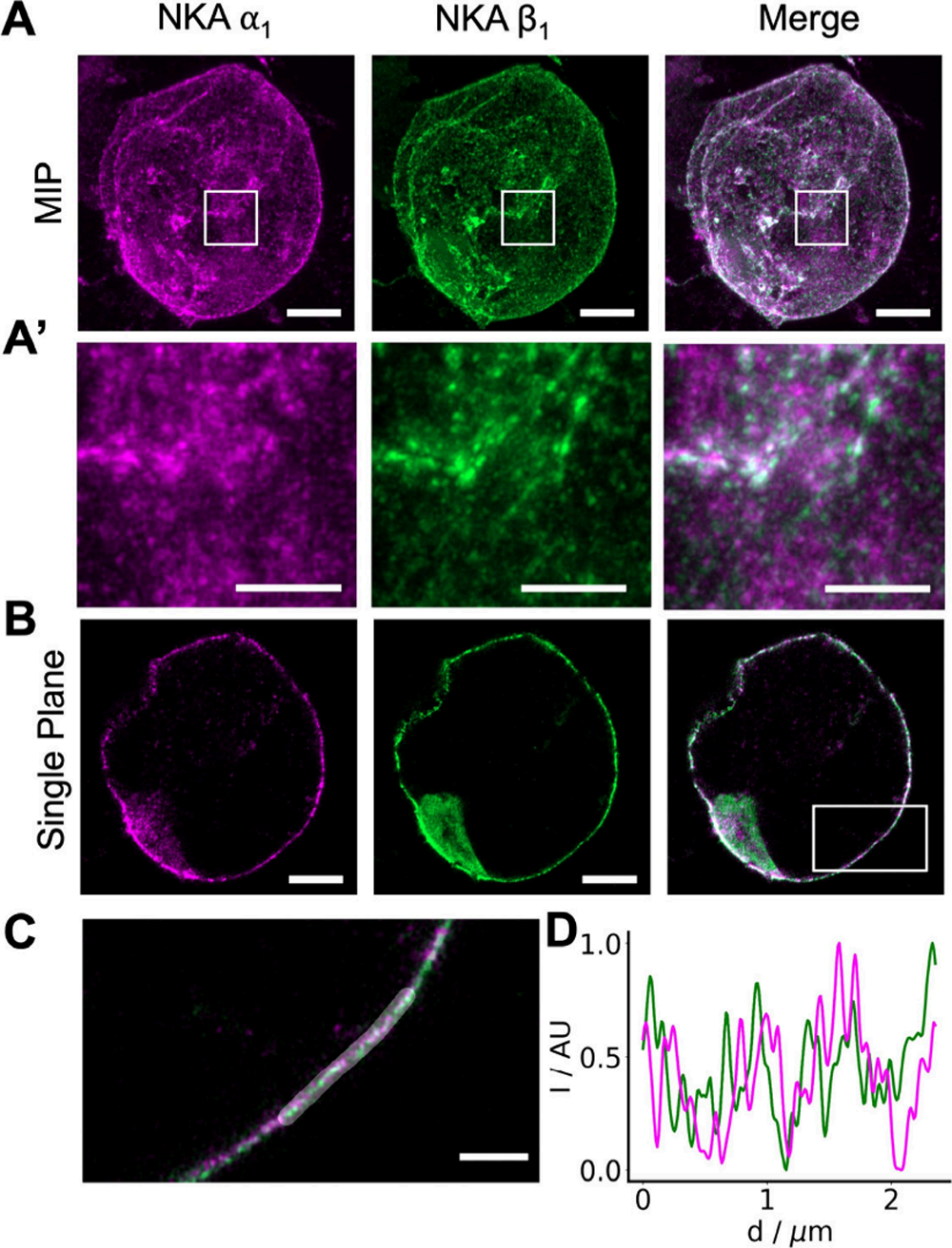
Two color biorthogonal labeling of NKA subunits α_1_ and β_1_ in expanded cells. Confocal z-stack (MIP) of an expanded HEK293T cell expressing NKA α_1_ T121TCO*K labeled with Abberior STAR 635-tetrazine (magenta) and β_1_ L64ProK labeled with AF488-picolylazide (green) (A). Boxed region from (A) shown in (A′). Single confocal plane through the center of an expanded HEK293T cell expressing NKA α_1_ T121TCO*K labeled with Abberior STAR 635-tetrazine (magenta) and β1 L64ProK labeled with AF488-tetrazine (green) (B). Boxed region from (B) shown in (C). Normalized line profile of highlighted membrane region in (C), showing heterogeneous distribution of NKA α_1_ (magenta) and β_1_ (green) (D). Scale bars: (A) and (B) 3 μm; (A′) and (C) 1 μm (all adjusted for expansion).

In summary we have developed and applied a workflow that combine GCE for site-specific labeling with ExM for super-resolution imaging. This approach enabled us to perform dual-color visualization of the NKA α_1_ and β_1_ subunits in HEK293T cells, providing nanoscale information on the enzyme’s distribution using a conventional confocal microscope. The primary motivation for this work is the fundamental challenge of determining the organization of proteins within the cell membrane. Many membrane proteins, including NKA, are proposed to exist not just as monomers, but also as dimers, higher-order oligomers, or organized in larger clusters.^2–6^ Distinguishing between these states is critical for understanding their function, but the nanometer-scale distances involved are far below the diffraction limit of conventional light microscopy. Therefore, resolving the true organization of such proteins demands super-resolution techniques that can achieve the highest possible localization precision.

A central advantage of GCE-based labeling is the minimization of “linkage error”. Common methods for fluorescence labeling using antibodies can add up to 20 nm of uncertainty to a protein’s position,^9^ a distance that can make it impossible to discern between a true dimer and two nearby monomers. Our approach reduces this to below 1 nm. Beyond labeling, the chemical fixation process itself poses a risk. Fixatives like paraformaldehyde (PFA) can induce artificial cross-linking of membrane proteins, potentially creating clusters that are not present in live cells.^20–23^ This possibility of fixation-induced artifacts must be considered when interpreting any super-resolution data on protein organization.

Stop codon suppression for fluorescent labeling is attractive, but also presents several challenges. Suppression of endogenous amber and ochre codons leads to read-through and ncAA insertion into unintended proteins, while inefficient suppression might result in truncated target proteins.^24^ In our case, the stop codon’s proximity to the mRNA’s 5′ end make it unlikely that truncated NKA subunits would fold correctly and be transported to the plasma membrane.

To avoid background from off-target ncAA incorporation, we used a membrane-impermeable dye to label extracellular domains. The validity of this strategy was confirmed with a control experiment. When we performed the click reaction after cell denaturation and permeabilization, we observed a bright, nonspecific intracellular staining that was present in both wild-type and ncAA-expressing cells. This confirms the presence of an intracellular off-target signal, possibly from nuclear labeling of charged tRNAs,^25^ and validates that our extracellular-only strategy was essential for obtaining a clean signal.

An important step for the quality of labeling was to ensure that the fluorescent labels could survive the chemical treatments of the ExM protocol. We found that the cyanine-based dye AF647-tetrazine was quenched by free radicals during gel polymerization. In contrast, AF488-tetrazine and the rhodamine-based dye Abberior 635-tetrazine were well-preserved throughout the expansion process. This illustrates that fluorophore stability must be empirically determined. To overcome quenching, it may be possible in the future to chemically modify sensitive dyes to protect them.^26^

GCE has been successfully combined with super-resolution methods like SMLM and STED to overcome the linkage error of antibody labeling.^27–30^ In our study, a 4.3-fold expansion combined with standard confocal microscopy provides an effective resolution of ∼60 nm, comparable to ncAA-STED. Using Airyscan detection this is further improved to 30–35 nm, approaching SMLM’s precision. While SMLM offers better localization, ExM can provide high-resolution imaging of dense 3D structures on standard hardware. Furthermore, combining ExM with STED has recently yielded resolution below 20 nm.^31^

While STED and ExM offer impressive resolution, it remains insufficient to definitively resolve individual NKA subunits within the plasma membrane, especially if the protein is highly clustered. Our expansion microscopy data revealed incomplete colocalization of the α_1_ and β_1_ subunits. Since both subunits are required for transport to the plasma membrane, we hypothesize that a proportion of the transfected, labeled subunits formed functional complexes with the pool of endogenous, unlabeled NKA subunits. This unlabeled pool needs to be considered when measuring protein cluster size from super-resolution data.

In conclusion, our work establishes a robust framework for multiplexed super-resolution imaging that addresses key challenges of labeling precision and fluorophore compatibility. Although limitations (such as the efficiency of ncAA incorporation and potential for minor expansion-induced distortions) exist, this method can be broadly applied to investigate the nanoscale organization of a wide variety of protein complexes using widely accessible confocal microscopes. Future refinements in expansion chemistry could further improve resolution, bringing angstrom-scale structural biology within the reach of fluorescence microscopy.

## Supplementary Material



## Notes

Additional experimental details, materials, and methods, including supplementary Figures S1–S3. The Supporting Information is available free of charge at https://pubs.acs.org/doi/10.1021/acs.nanolett.5c05301.
