# Supplementary material for: Dual-Color Expansion Microscopy of Membrane Proteins Using Bioorthogonal Labeling
Source: Nano Lett. 2026 Jan 22;26(4):1321–6. doi: 10.1021/acs.nanolett.5c05301 (PMC12879918; doi:10.1021/acs.nanolett.5c05301)
Supplement: Supplementary file 1 [file nl5c05301_si_001.pdf]

Supporting information for

Dual-color expansion microscopy of membrane proteins using bioorthogonal labeling

Steven Edwards[1], Birthe Meineke[2], Sebastian Bauer[2], Hans Blom[1], Simon Elsässer[2], Hjalmar Brismar[1,2,]*

[1]Science for Life Laboratory, KTH Royal Institute of Technology, Sweden

[2]Science for Life Laboratory, Karolinska Institute, Sweden

[*]Correspondence should be addressed to Hj.B. (brismar@kth.se)

I - Materials and Methods

II - Additional figures

I. **Methods**

Cell culture

HEK293T cells were passaged every 3-4 days and cultured at 37°C, 80% humidity and 5% CO2 in Dulbecco's modified Eagle's medium (DMEM, Sigma-Aldrich) media supplemented with 10% FBS and 1% penicillin-streptomycin. For transfection, cells were seeded at a density of 25 000 cells/cm^2 onto 10 mm diameter #1.5 coverslips pre-coated with Poly-L-Lysine (P4707; Sigma-Aldrich). Cells were allowed to grow for 24 hours prior to transient transfection.

DNA constructs and transfection

Plasmids for EF1 promoter-controlled expression of the NKA α_1 and β_1 subunits (rat) containing amber (TAG) or ochre (TAA) stop codons were generated based on pAS (#140008 Addgene). Point mutation for α_1 T121TAG, β_1 L64TAA and β_1 L64TAG were introduced by two step PCR from the wild type sequences [3]. To facilitate dual stop codon suppression the four tandem h7SK-PylT repeats were exchanged for a total of eight tRNA genes: four tandem repeats coding for *Mx1201/G1* hybrid $tRNA^{Pyl}{}_{CUA}$ A41AA C55A (hyb*) controlled by h7SK promoter, and four tandem repeats coding for U6 promoter controlled *Mma* $tRNA^{Pyl}$ variant M15 with a UUA anticodon ($M15_{UUA}$). The two aminoacyl-tRNA synthetases (aaRS) used were pAS_4xhybPylT A41AA C55A FLAG-G1 PylRS Y125A (#154773; Addgene) for amber suppression and pAS_4xU6-PylT M15 (UUA) FLAG-Mma PylRS (#154774; Addgene) for ochre suppression [17].

Cells were transiently transfected with 500 ng DNA/cm^2 with a 9:1 NKA α/β 1:aaRS ratio. TransIT-LT1 (MIR2305; Mirus) transfection reagent was used with Opti-MEM I reduced serum medium (31985062; Thermo Fisher Scientific) according to manufacturer's instructions. In single labeling experiments, a WT NKA α_1 or β_1 subunit was co-transfected (1:1) with the mutated subunit.

The ncAAs axial trans-cyclooct-2-ene–lysine (TCO*K, #SC-8008; SiChem) and N-Propargyl-L-lysine (ProK, #HAA2090; Iris Biotech) were added to the cell culture media at the same time as transfection, the cells were then grown for 48 h to allow sufficient protein expression prior to labeling and fixation. Unless stated otherwise, final concentrations were 0.25 mM ProK and 0.1 mM and TCO*K, respectively.

Click Chemistry

ProK was labeled using Cu(I)-catalyzed azide-alkyne cycloaddition (CuAAC) reaction using 1 µM dye AF647/488-picolyl azide (Jena Bioscience) in a buffer containing 50 µM $CuSO_4$

(Copper(II) sulfate pentahydrate, #209198; Sigma-Aldrich), 250 μM THPTA (Tris(3-hydroxypropyltriazolylmethyl)amine, #762342; Sigma-Aldrich) and 2.5 mM L-ascorbic acid (#A5960; Sigma-Aldrich) in PBS [18]. $CuSO_4$ and THPTA were vortexed together and kept on ice for 5 minutes before adding freshly dissolved L-Ascorbic acid, dye, and incubated for 10 minutes on ice. The reaction mixture was added to cells, and the reaction was allowed to proceed at room temperature for 5 minutes on live cells, 30-60 minutes on fixed cells, and 3 hours on expanded gels.
TCO*K was labeled by using strain-promoted inverse electron-demand Diels-Alder cycloaddition (SPIEDAC) with 1 μM AF488/647-tetrazine (Click Chemistry Tools) or Abberior STAR 635/STAR RED-tetrazine (Abberior GmbH). For live cells, the dye was added directly to the PBS, and the cells were incubated for 30 minutes at 37°C. For fixed cells, the dye was mixed with PBS, and the cells were incubated at RT for 1 hour. For expanded gels, the dye was mixed in PBS, and the gels were allowed to incubate for 3 hours at room temperature.

Expansion

Cells were fixed in 4% paraformaldehyde (PFA) for 15 minutes at RT prior to incubation in a humidified chamber at 37°C in AA/FA solution (30% Acrylamide (AA) and 4% Formaldehyde (FA)). Cells were washed 3x5 min in PBS. 35 μL of monomer solution (7% sodium acrylate (SA), 20% AA, 0.05% N,N'-methylen-bisacrylamide (Bis-AA), 0.5% Ammonium persulfate (APS), and 0.5% N,N,N′,N′-Tetramethylethylenediamine (TEMED) in PBS was placed on glass slide covered with parafilm. The slide was placed on ice to create a cooled hydrophobic gelation surface. The coverslip was placed upside down onto the monomer solution and left for 5 minutes on ice before being moved to a humidified chamber at 37°C for 1.5 hours to polymerize. This coverslip and gel were removed from the parafilm and dropped into a denaturation buffer (200nM SDS, 200 mM NaCl, and 50 mM TRIS, pH 9.0) at 95°C for 1 hour. The gels were allowed to fully expand by washing them several times in deionized water. The diameter of the fully expanded gel was measured and divided by the coverslip diameter (10 mm) to calculate the expansion factor.

Mounting

Fully expanded gels were mounted on a glass-bottom dish (P35G-1.5-20-C; MatTek Corporation) precoated with Poly-L-Lysine (P4707; Sigma-Aldrich). Gels were covered with 1.5% UltraPure low melting point agarose (Invitrogen) in deionized H_2O to support the gel during imaging. After polymerisation of the agarose, the dish was filled with deionized H_2O.

Imaging

Images of expanded gels were acquired on a Zeiss LSM 980 with Airyscan detection using a 40 X 1.2 NA water immersion objective. Images of fixed cells (Figure 1) were acquired using a Zeiss LSM 980 confocal with a 63 X 1.4 NA oil immersion objective. Confocal and STED images of fixed cells (Figure 2) were acquired using a using a Leica SP8 3X microscope with a 100 X 1.4 NA oil immersion objective.

Image analysis

To determine the size of individual NKA clusters, we identified local intensity maxima within the fluorescence images. The two-dimensional (2D) intensity profile surrounding each maximum was then fitted to a 2D Gaussian function. We defined the cluster diameter as the Full Width at Half Maximum (FWHM) of the resulting Gaussian fit.

SDS-PAGE and western blot

Cells were washed and collected in phosphate-buffered saline (PBS). Lysis was performed on ice using PBS supplemented with 0.2% triton X-100 (v/v) and 1× complete protease inhibitor cocktail (Roche). Lysates were cleared by centrifugation, mixed with 6× Laemmli buffer and denatured for 10 min at 37 °C. Protein expression was confirmed by standard western blotting using the following primary antibodies: anti-HA-Peroxidase, rat monoclonal antibody (Roche Cat# 12013819001, RRID:AB_390917) for HA-fusion proteins, anti-Na+/K + -ATPase alpha-1 subunit, mouse monoclonal antibody (Developmental Studies Hybridoma bank Cat# a6F, RRID: AB_528092) for α1; and anti-Na+/K + -ATPase beta-1 subunit, mouse monoclonal antibody (Santa Cruz Biotechnology Cat# sc-21,713, RRID:AB_626714) for β1.

II – Supplemental figures

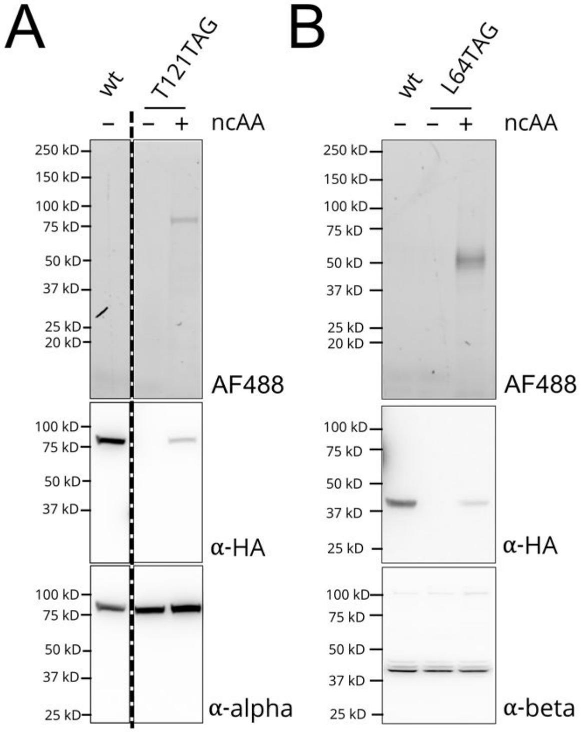

Figure S1: Immunoblot analysis confirming expression of $Na^{+}K^{+}$-ATPase subunits and HA-tagged fusion protein.

Cell lysates were prepared using PBS supplemented with 0.2% Triton X-100 and protease inhibitors. Following clarification and denaturation, proteins were separated by SDS-PAGE and transferred for Western blotting. Expression was confirmed using AF488 fluorescence to detect incorporation of the ncAA, and primary antibodies targeting HA-fusion proteins (anti-HA-Peroxidase, Roche Cat# 12013819001), the endogenous $Na^{+}K^{+}$-ATPase alpha1 subunit (anti-alpha1, DSHB Cat# a6F), and the endogenous $Na^{+}K^{+}$-ATPase β_1 subunit (anti-beta1, Santa Cruz Biotechnology Cat# sc-21713).

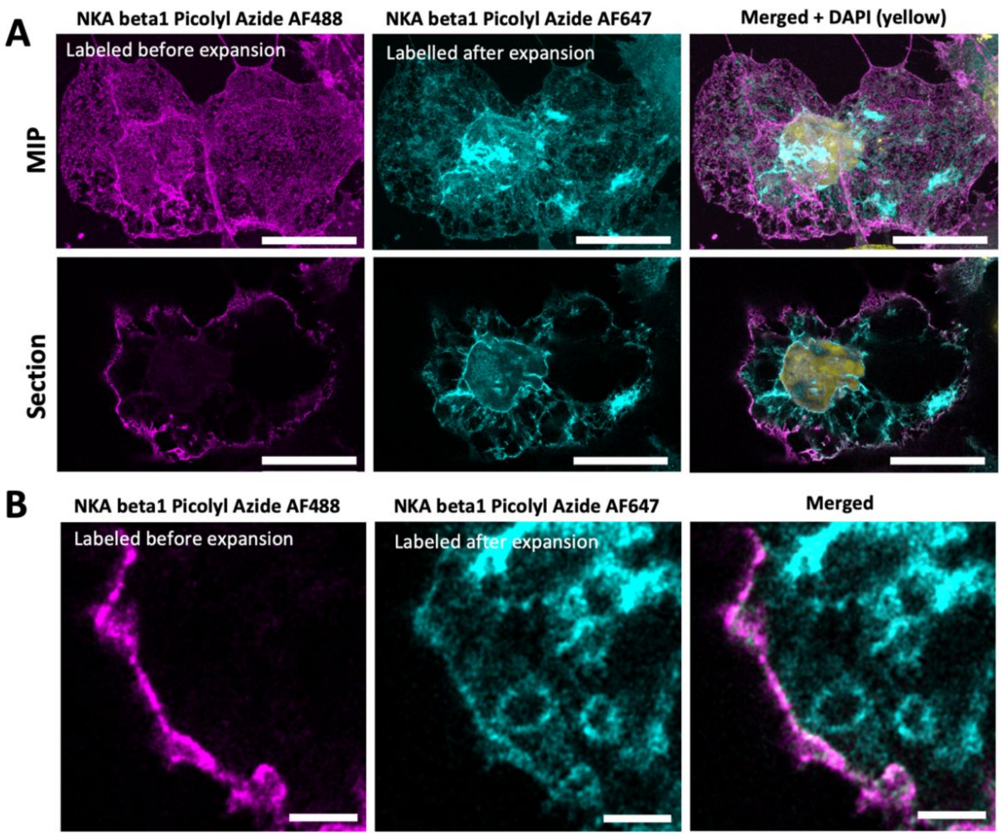

Figure S2: CuAAC labeling can be performed after denaturation to label intracellular ncAAs. Confocal image of an expanded HEK293T cell expressing NKA β_1 L64ProK labeled before fixation with AF488-picolyl azide (magenta) and after denaturation with AF647-picolyl azide (cyan), top row: MIP, bottom row: sing section **(A)**. Zoomed confocal image of an expanded HEK293T cell expressing NKA β_1 L64ProK labeled before fixation with AF488-picolyl azide (magenta) and after denaturation with AF647-picolyl azide (cyan) (**B**). Scale bar (A): 10 µm, scale bar (B) 4 µm (adjusted for expansion).

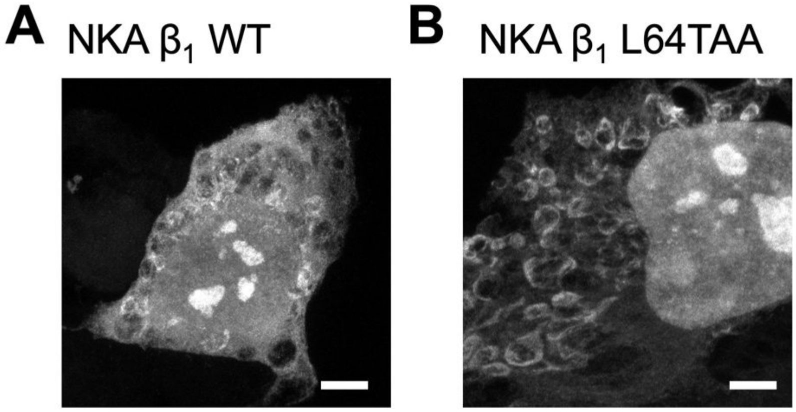

Figure S3: Post-denaturation labeling of both NKA β_1 WT and β_1 L64TAA transfected cells shows intracellular structures.

Confocal stack (MIP) of HEK293T cells transfected with $M15_{UUA}$/*Mma*PylRS and NKA β_1 WT, grown with ProK, expanded, and CuAAC labeled with AF488-picolyl azide after denaturation (A). Confocal stack (MIP) of HEK293T cells transfected with $M15_{UUA}$/*Mma*PylRS and NKA β_1 L64TAA, grown with ProK, expanded, and CuAAC labeled with AF488-picolyl azide after denaturation (B). Scale bar 10 μm.
